# Notes

**Published:** 1899-08

**Authors:** 


The committee of arrangements for the Scranton meeting of the Pennsylvania State Veterinary Medical Association meeting, appointed by President J. Curtis Michener, are as follows: Dr. Jacob Helmer, Scranton; Dr. James Hardin Perman, Wilkesbarre; and Dr. John R. Courtwright, Clark's Green. The reception committee: Drs. Jacob Helmer and C. S. Gelbert, of Scranton.

The delegates of the Pennsylvania Association to the American Veterinary Medical Association at New York will also have many others of the State Association in attendance.

New Jersey will be represented at New York, and will add several new members from her number. When will the veterinarians of the "Jersey Blue State" get together in one strong organization and work together for better recognition in their own State, readjust legislation of a veterinary sanitary character along proper lines, that will bring to her people and interests what other commonwealths are reaping?

### NOTES.

The reports on the results of iodide of potassium treatment in milk-fever, or parturient apoplexy, continue to be more favorable than any previous line of treatment so generally adopted.

The Indianapolis Board of Health is making earnest and successful warfare on the milk-dealers using formaldehyde as a preservative. Proofs of its injurious influences on digestion of man and child have been the groundwork of the prosecution.

Several veterinarians recently failed in the examination for army service on the general education qualification. This requirement is stated to have been about the same as entrance examination to West Point.

Several of Allegheny County's veterinarians pay little respect or acknowledgment to the code of ethics in advertising, and set a bad example for younger practitioners in that territory. Associations that may have jurisdiction should look after their erring members at that point.

Anthrax among sheep in the neighborhood of Manchester, Mich., is reported, and prompt action is urged upon the authorities to use such sanitary police measures as shall limit its ravages.

Dr. James A. Waugh reports the condemnation of forty-nine cows out of a herd of sixty recently tested in Washington County, Pennsylvania.

The staff of the University of Nebraska are studying some plan to deal successfully with the prairie-dog nuisance, in the direction of their extermination. Poison, inoculation with certain specific diseases, are being tried and reported upon, and the dangers, time required, etc., are all carefully noted.

The Humane Society and the Genesee Valley Veterinary Medical Association have issued statements denouncing certain self-styled "dental specialists" who have advertised that they are highly recommended by the societies named. Both societies disclaim any knowledge of the "specialists," and both insist that they have not recommended them, and, furthermore, are very much opposed to their methods.

